# Liquid metal injected from interstitial channels for inhibiting subcutaneous hepatoma growth and improving MRI/MAT image contrast

**DOI:** 10.3389/fonc.2022.1019592

**Published:** 2022-11-21

**Authors:** Chaosen Lu, Aocai Yang, Fei Xia, Guoqiang Liu, Hongliang Zhao, Wenwei Zhang, Yuanyuan Li, Jian Liu, Guolin Ma, Hui Xia

**Affiliations:** ^1^ Department of Engineering Electromagnetic Field and Its Application, Institute of Electrical Engineering Chinese Academy of Sciences, Beijing, China; ^2^ College of Electrical and Automation Engineering, Shandong University of Science and Technology, Qingdao, China; ^3^ Department of Radiology, China-Japan Friendship Hospital, Beijing, China; ^4^ Graduate School of Peking Union Medical College, Chinese Academy of Medical Sciences and Peking Union Medical College, Beijing, China; ^5^ Artemisinin Research Center, Institute of Chinese Materia Medica, China Academy of Chinese Medical Sciences, Beijing, China; ^6^ Department of Electronic and Electrical Engineering, University of Chinese Academy of Sciences, Beijing, China

**Keywords:** liquid metal, hepatoma, magnetic resonance imaging, magnetoacoustic tomography, interstitial injection

## Abstract

**Objective:**

Liquid metal (LM) nowadays is considered a new biomedical material for medical treatment. The most common application of LM in medical therapy is taking LM as a carrier for oncology therapeutics. However, the feasibility and direct effect of LM in tumor treatment are still unknown, and how to delineate the negative resection margin (NRM) of the tumor is also a crucial problem in surgery. We aimed to inject LM into interstitial channels of extremities of mice to overlay the surface of the primary tumor to investigate the effect of LM on inhibiting tumor growth and highlight the NRM of the tumor.

**Methods:**

In this study, all 50 BALB/c-nude female mice were used to construct the transplanted HepG2-type hepatocellular carcinoma model. One week after the establishment of the model, the mice were divided into three groups, named LM group, PBS group and Control group by injecting different liquid materials into the forelimb interstitial channel of the mice. T2WI image on MRI and Magneto-acoustic tomography (MAT) were used to show the distribution of LM and PBS in vivo. The group comparisons of tumor growth and blood tests were evaluated by one-way ANOVA and post-hoc analysis. And the biocompatibility of LM to BALB/c nude mice was evaluated by histopathological analysis of LM group and control group.

**Results:**

The volume change ratio of tumor was significantly lower in LM group than in PBS and Control group after 10 days of grouping. Compared with PBS and Control group, the main indexes of blood tests in LM group were significantly lower and close to normal level. In addition, the distribution of LM in vivo could be clearly observed under T2WI anatomic images and the crossprofile of the tumor in MAT. LM also has a obvious contrast in MRI T2WI and enhanced the amplitude of imaging signal in MAT.

**Conclusion:**

LM may inhibit the growth of transplanted hepatoma tumor through tumor encapsulation. In vivo, tumor imaging and LM distribution imaging were achieved by MRI T2WI, which verified that LM injected with interstitial injection made the NRM of tumor more prominent and had the potential of being MRI contrast agent. At the same time, LM could also be a new conductive medium to improve the imaging quality of MAT. Moreover, LM performed mild biocompatibility.

## Introduction

Hepatocellular carcinoma is the most prevalent of primary tumors and a common malignant tumor (1), which greatly threatens human health. With the development of a therapeutic strategy, the current therapy of primary hepatocellular tumor mainly depends on the comprehensiveness of treatment (2, 3), which includes surgery, interventional therapy, targeted therapy, and immune therapy (4). A gallium-based alloy is an admixture with liquid status under room temperature and is high profile in biomedical engineering fields due to its excellent properties encompassing bio-compatibility, controllability (5, 6), high conductivity, and good thermal conductivity (7, 8).

With deepening research on the gallium-based alloy, many studies show the merits and applications of liquid metal (LM). LM as a novel functional material has gradually gained attention in biomedical materials, wearable electronics, etc. (9, 10). Regardless if it is a nanodrug carrier for combined oncology treatment (11) or a new nonmagnetic heat therapy medium for thermal oncology (12, 13), LM as an interventional vehicle for combined tumor therapy has been a rising trend. Previous studies indicate that LM possesses excellent liquidity in the fascia space of the interstitial structure and the possibility of effectively transmitting LM to the target area in specific depth. The above research also provides a new insight for carcinoma combination therapy. At present, early observation and complete resection is main method of treatment for early stage primary tumor. Magnetic resonance imaging (MRI) and computer tomography (CT) are the mainstream methods for observing the primary tumor to illustrate the exact position of the tumor in the body and indicate the negative resection margin (NRM) of the tumor (14, 15). It is difficult for conventional MRI to define the tumor’s NRM, so it is important to seek a new method to improve the tissue contrast of MRI for the tumor’s NRM (16). For example, to clearly display the tumor’s NRM in MRI, it is feasible to add the MRI contrast agent or imaging medium without hydrogen. Similarly, LM with high conductivity is an excellent detectable medium for magnetoacoustic tomography with magnetic induction (MAT) (17), in which the main detection target of MAT is the conductivity distribution in tissues *in vivo*. However, the signal-to-noise ratio (SNR) of detection signal is always low due to the low of target tissues. In order to effectively obtain the magneto-acoustic signals with high SNR, injecting LM into the detected tissue to improve the intensity of the magneto-acoustic signal was proposed (18). Furthermore, we previously proposed a method to locate the spatial distribution of LM using the envelope of the magneto-acoustic signal collected in a circle (19). Therefore, in the field of biological tissue electrical impedance imaging technology, LM has broad application potential as MAT signal enhancement medium or conductivity comparison medium.

In this study, we hypothesized that the interstitial space is an effective transmission channel for LM to arrive at a specific depth *in vivo* and that the presence of tumors disrupts the closed interstitial channels, causing LM to accumulate on the tumor surface. The spatial distribution and geometric shape of tumor in tumor-bearing mice were observed by MRI T2WI image. And, we evaluated the enhancement effect of LM on tumor image by MRI T2WI and MAT. In addition, in order to confirm the biological safety of LM in this study, the potential physical influence of this method was evaluated with the combination of histopathological, hematological and serum biochemical analyses.

## Materials and methods

### Animal model of HepG2 hepatocellular carcinoma

All animal experiments and implementing measures were approved by the Biomedical Research Ethics Review Committee of Institute of Electrical Engineering, Chinese Academy of Sciences.

A gallium-based alloy composed of 75% gallium and 25% indium was from Alfa Aesar (China) chemical Co., Ltd. (Shanghai, China). Female BALB/c-nude mice (with body weight of approximately 20 g) were from the department of laboratory animal science of Peking University Health Science Center (Beijing, China). HepG2 hepatocellular carcinoma cells (HepG2 cells) were from the National Bio-medical laboratory cell repository (Beijing, China).

HepG2 cells were cultured in a medium supplemented with 10% fetal bovine serum (FBS), 100 ug mL-1 penicillin, and 1 mg mL-1 streptomycin at 37°C in an incubator mixed with 95% air and CO_2_. HepG2 cells (3.0 × 106/0.2 mL) suspended in PBS were subcutaneously injected into the right upper extremity of each female BALB/c-nude mouse. HepG2 cells were derived from well-growing tumors of tumor-bearing mice, which were separated from the newly disposed tumor-bearing mice by alcohol disinfection under aseptic conditions. Tumor masses with necrotic tissue removed were weighed, cut, and ground ina glass homogenizer and diluted into cell suspension at a ratio of 1:3 with normal saline in which the number of cells approximately was 3.0 × 106/0.2 ml. The cell suspension was injected into the subcutaneous tissue of BALB/C-nude female mice and fed 1 week to get the animal model of HepG2 Hepatocellular carcinoma.

### Experiments design

A total of 50 tumor-bearing mice participated in the experiment. Hepg2-type hepatocellular carcinoma model. When the tumor model was established for one week, they were divided into LM group, PBS group and Control group by injecting different fluid media into the interstitial channel of the forelimb of mice, respectively. The number of BALB/C-nude female mice in LM group was 18 and that in PBS group and Control group was 16, respectively. Mice in LM group were injected with 1mL LM. Mice in PBS group were injected with 1mL PBS and mice in control group had natural tumor growth without any operation. The begin time of experiments was defined on the day of grouping which was named as Day 0 on recording time. All these selected mice were used to observe the distribution and size change of tumor on MRI and MAT platform. Four mice in each group were randomly selected for histopathological analysis and blood test, which were performed at the time points defined as Day0, Day3, Day6 and Day12.

### MRI data acquisition and tumor observation

A 3.0 T MRI scanner (GE, Discovery MR750, Milwaukee, United States) with a standard animal coil (Shanghai Chenguang Medical Science and Technology, Shanghai, China) was used to acquire the data. All mice were anesthetized with 3% sodium pentobarbital (1.3 uL/g) by intraperitoneal injection before scanning (20). The mice were placed in the prone position in the scanning coil and fixed with a vacuum pillow and strap to reduce the movement of the body. T2WI anatomic images were reconstructed using Fast Spin Echo sequences (FSE), and three positional images were all scanned, including axial, coronal, and sagittal positions. T2WI-FSE data was acquired with the following parameters: repetition time (TR) = 3225 ms, echotime (TE) = 81 ms (sagittal)/86.9 ms (coronal)/88.7 ms (axial), flip angle = 111°, voxel size = 0.16 × 0.16 mm^2^, number of slices = 12–15, slice thickness = 1.5 mm.

In order to observe the distribution of LM *in vivo* and the tumor’s variation and assess the imaging ability of LM in MRI, we segmented the tumor and LM by manually delineating the boundary of the tumor and LM in continuous slices of T2WI images. Finally, the volumes of segmented tumors and LM were automatically calculated by ITK-SNAP based on the voxel size and slice thickness of T2WI images. The above operations were performed using ITK-SNAP software (http://www.itksnap.org).

### Histopathological analysis

This analysis was mainly used to assess the potential biological toxicity of LM toward female nude mice. Major organ slices, including hearts, livers, spleens, kidneys, and tumors were harvested from the mice of each group. Organ slices were put into solution, mixed with 10% neutral buffered formalin solution for 12 h, and stained with hematoxylin and eosin and dehydrated by 75%–100% anhydrous ethanol. The samples were examined in a blinded manner by a well-trained pathologist, organs were examined with an average of five fields of view, and histological microscopy was finished with a digital slide scanner.

### Hematology and serum biochemical tests

Blood tests were mainly used to detect the expression level of abnormal indexes in blood caused by the injury of organs. The method of eyeball extraction was used in each mouse to abstract a blood specimen on account of low weight and small size. Blood samples (>170 uL) wwere put into centrifugal tubes, harvesting serum by refrigerated centrifuge (Thermo Fisher, Fresco21) with 5000 r/min speed for 7 min. Hematology and serum biochemical tests were accomplished by Toshiba 120 fully automatic analyzer. Alanine aminotransferase (ALT), aspartate aminotransferase (AST), creatinine (CREA), and carbamide (UREA) are used for evaluating the injury of liver and/or kidney, and white blood cell count (WBC), red blood cell count (RBC), hemoglobin (HGB), and platelet count (PLT) were used for analyzing the pathological change of hematopoietic organs.

### Enhancement of LM for MAT signal

During the experiments, we utilized MRI and MAT images to observe the distribution of LM in vivo and tumor growth, and evaluated the effect of LM in improving image quality. In order to study the image enhancement effect of LM on MAT, based on the MAT imaging platform system developed by our team, we first prepared a cylindrical agar model with gaps. By injecting liquid media with different conductivity into the gaps, we verified that LM can improve the weak detection signal of MAT and enhance the imaging ability. Then, BALB/c-nude mice were used to construct the living animal tumor model, and the enhancement effect of LM on the weak signal of MAT and the image enhancement effect of tumor in vivo were further verified by injecting LM. The liquid materials such as PBS, Deionized water (DI), Gallium-based alloy Microparticles (GMs) and LM were filled into the gap reserved in the cylindrical agar model to simulate the phenomenon of liquid materials wrapping tumors. Magneto-acoustic tomography experiments were carried out on cylindrical agar phantom models filled with different liquid materials. Magneto-acoustic distribution images of different liquid materials were obtained by extracting the magneto-acoustic signal intensity of each channel of MAT, and the image contrast of different liquid materials in the agar model was observed. At the same time, the mice injected with LM were studied by magneto-acoustic tomography to observe the distribution of LM in vivo.

### Statistical analysis

The vernier caliper and T2WI images were used to obtain the tumor size of each mouse in every group. The volume can be approximated by b2 × a× 0.5, where a and b represent the length and width of the tumor. The tumor volume changes ratio of each group across days was calculated based on the tumor volume of Day0. All the statistical analyses were performed on GraphPad Prism 8 (GraphPad Software, San Diego, CA, USA). Group differences in continuous variables were evaluated by one-way ANOVA and post-hoc analysis. Tukey's method was used for multiple comparison correction. Group comparisons among four groups included tumor volume change rate across growth time, serum biochemical indexes and hematological indexes. Significant threshold was set at P<0.05.

## Results and discussion

### Inhibition effect of LM for tumor growth

**Figure 1:**
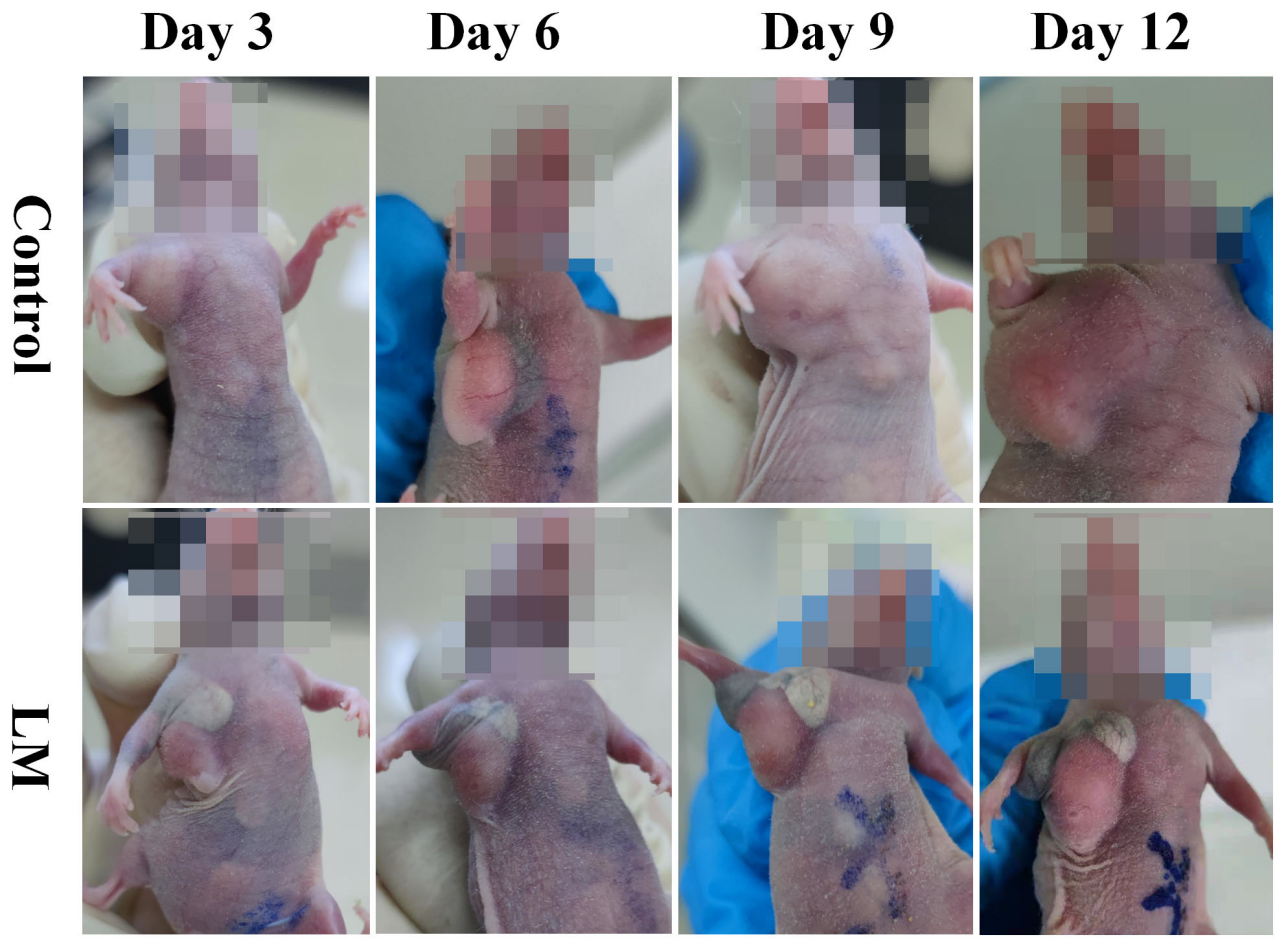
The morphological variation of tumors in LM group and Control group on different growth days. Eight mice were selected in each group to record tumor’s growth. LM, liquid metal.

**Figure 2:**
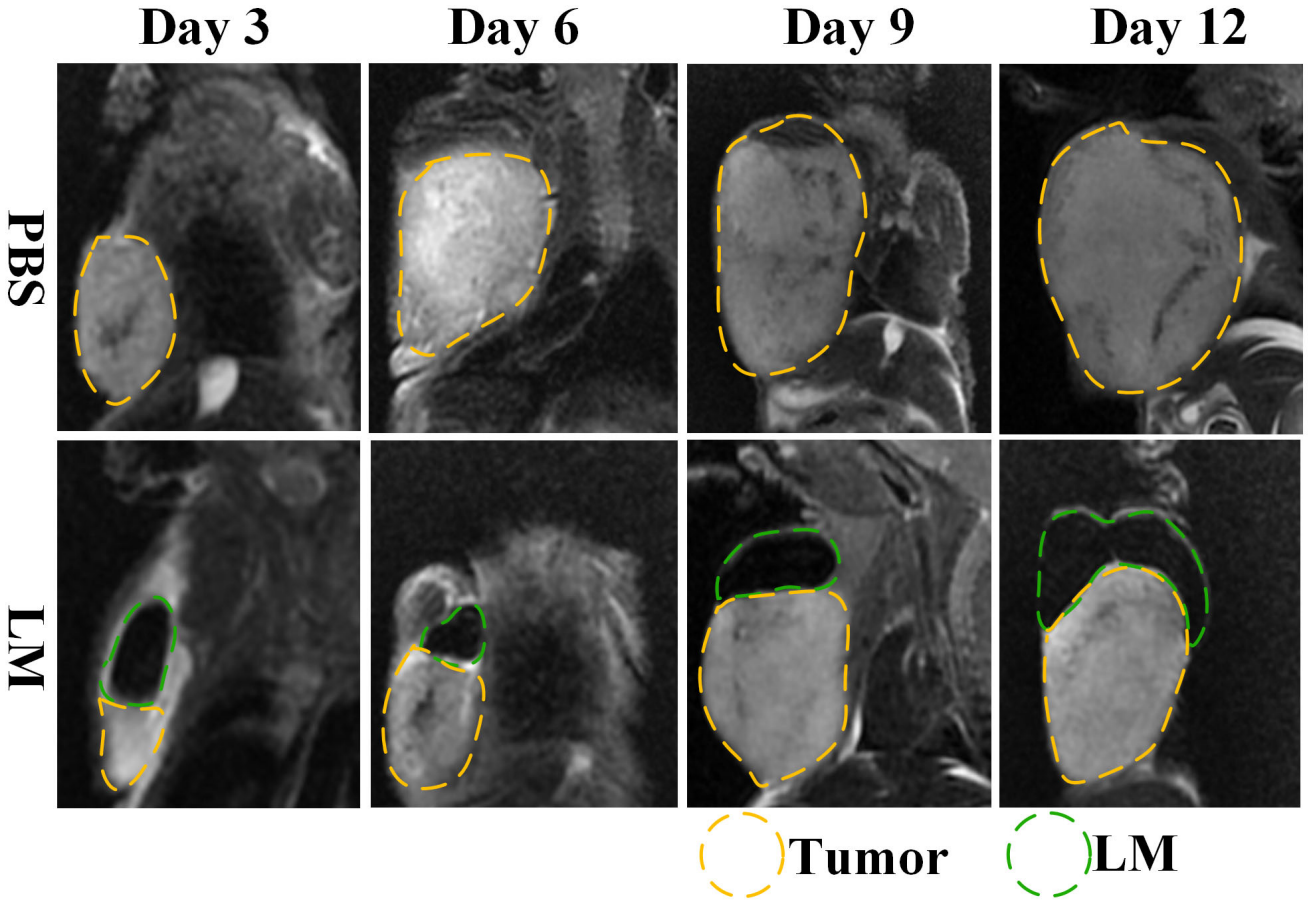
MRI T2WI images of PBS group and LM group on different growth days. The LM coverage area is marked with a green dotted line, and the tumor area is marked with a yellow dotted line. LM, liquid metal; PBS, phosphate buffer saline.

The morphological variation of tumors of tumor-bearing mice in the LM and Control groups are displayed at different times on **Figure 1**. As shown in **Figure 1**, the tumor growth rate of the LM group was obviously lower than other groups, and the distribution of LM in the body can be seen directly. The T2WI anatomic images of the PBS and LM groups at different time are shown in **Figure 2**. On the Day6, the tumor region in the PBS group was brighter than other tissue region. However, it should also be seen that PBS was absorbed faster in the body. Compared with the PBS group, T2WI image in LM group showed the tumor morphology more obviously. Because of the LM injection, the boundary of the tumor was more distinguished.

**Figure 3:**
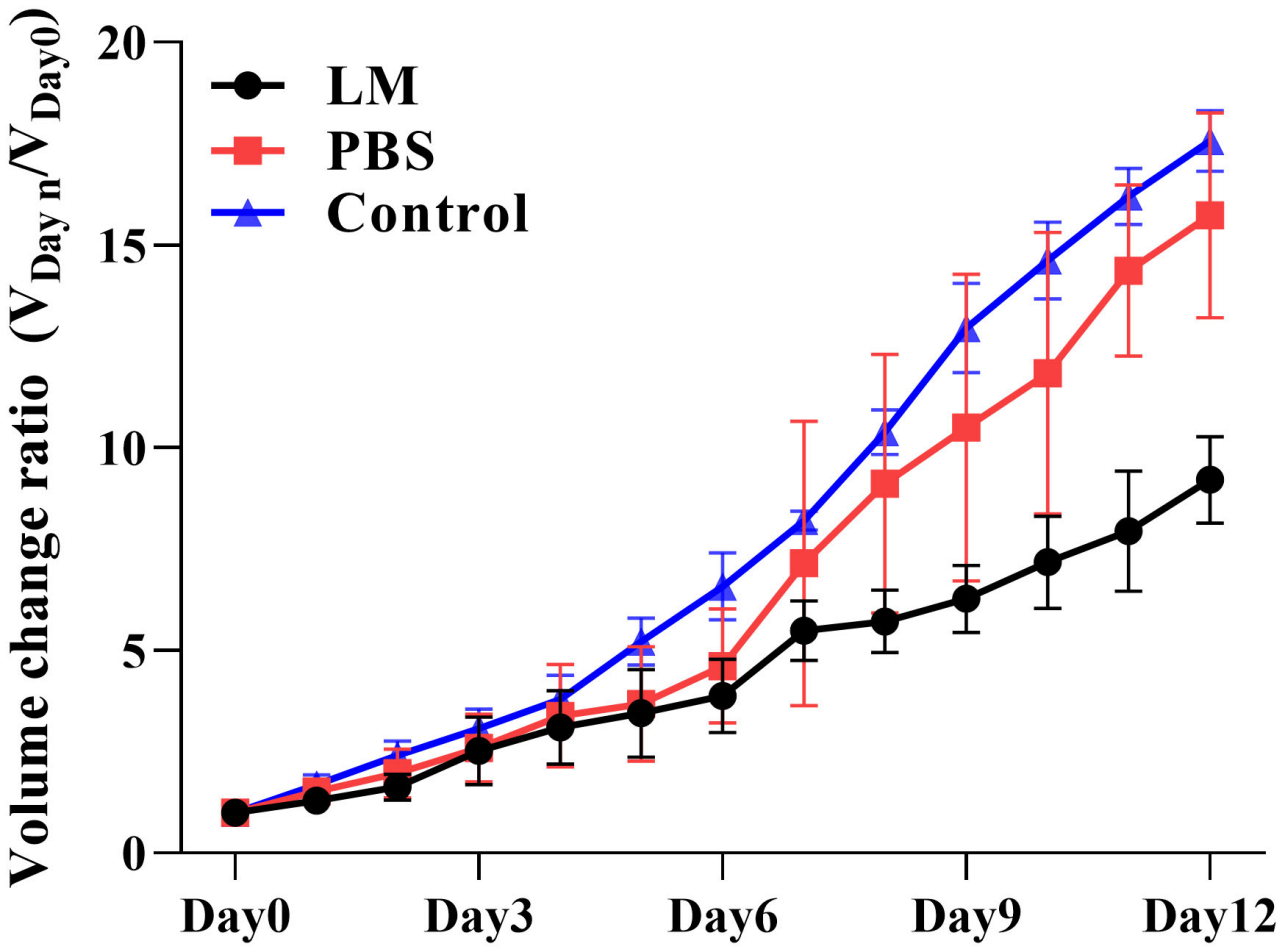
Trend plot of tumor volume ratio with different growth days in LM group, PBS group and control group. V*_Dayn_*/V*_Day0_* represents the change rate of tumor volume on day n relative to that on day 0; V*_Day0_* represents the tumor volume on day 0. Eight mice were selected in each group to measure the tumor size every day. LM, liquid metal; PBS, phosphate buffer saline.

The trends of tumor volume change ratio with days in each group was shown in **Figure 3**. The tumor growth rate in LM group was significantly lower than that of PBS group and Control group. **Table 1** showed the statistical results of the volume change ratio of primary tumor across days among three groups and **Table 2** was the results of post-hoc analysis corresponding with **Table 1**. Combined with the analysis in **Table 2**, there were statistically significant differences between LM group, PBS group and Control group.

**Table 1:** The statistical results of the volumes change ratio of primary tumor across days among three groups.

	LM Group(n=8)	PBS Group(n=8)	Control Group(n=8)	One-way ANOVA	
	Mean±SD			F value	P value
**Day1**	1.292±0.298	1.523±0.296	1.687±0.234	2.046	0.1852
**Day2**	1.627±0.316	1.964±0.603	2.404±0.353	3.101	0.0946
**Day3**	2.520±0.828	2.592±0.836	3.075±0.477	0.676	0.5325
**Day4**	3.091±0.904	3.387±1.260	3.785±0.594	0.528	0.6072
**Day5**	3.444±1.080	3.679±1.407	5.216±0.584	3.188	0.0898
**Day6**	3.875±0.905	4.610±1.405	6.577±0.830	6.726	0.0163*
**Day7**	5.488±0.732	7.142±3.511	8.199±0.229	1.735	0.2304
**Day8**	5.715±0.773	9.111±3.187	10.380±0.547	6.320	0.0193*
**Day9**	6.268±0.831	10.500±3.781	12.950±1.101	8.460	0.0086**
**Day10**	7.174±1.138	11.830±3.472	14.620±0.948	11.910	0.0030**
**Day11**	7.940±1.480	14.360±2.109	16.190±0.690	28.770	<0.0001***
**Day12**	9.206±1.068	15.730±2.521	17.570±0.747	31.690	0.0001****

LM, liquid metal; PBS, phosphate buffer saline; SD, standard deviation; n, number of sample; *, indicates P<0.05; **, indicates P<0.01; ***, indicates P<0.001; ****, indicates P<0.0001.

**Table 2:** The statistical results of post-hoc analysis for the volumes change ratio of primary tumor across days.

	LM vs. PBS	LM vs. Control	PBS vs. Control
	Adjusted P value		
**Day1**	0.4953	0.1646	0.6907
**Day2**	0.5510	0.0808	0.3780
**Day3**	0.9894	0.5550	0.6358
**Day4**	0.9016	0.5815	0.8301
**Day5**	0.9496	0.1032	0.1637
**Day6**	0.6156	0.0155*	0.0693
**Day7**	0.5220	0.2091	0.7581
**Day8**	0.0784	0.0183*	0.6328
**Day9**	0.0702	0.0071**	0.3394
**Day10**	0.0348*	0.0024**	0.2219
**Day11**	0.0006***	<0.0001****	0.2643
**Day12**	0.0008***	0.0001***	0.2994

LM, liquid metal; PBS, phosphate buffer saline; Adjusted P value, the statistical result of Tukey's multiple comparisons test; *, indicates P<0.05; **, indicates P<0.01; ***, indicates P<0.001; ****, indicates P<0.0001.

Furthermore, **Figures 2**, **3** also showed that the LM on the tumor surface has an effective blocking effect on tumor nutrient supply, blocking tumor nutrient supply and isolating the tumor from healthy tissues. However, it was also seen that there were some omission areas of blocking effect on the surface of tumor, which may cause the tumor growth in the direction of the non-blocking region. It was noteworthy that the increased trend of tumor in LM group was significantly lower than that in the PBS group and Control group from the Day 8 in **Figure 3** and **Table 2** (F=6.320, P=0.0193 at Day 8; Post-hoc analysis: LM vs. PBS, P<0.048* at Day 8; LM vs. Control, P=0.0183 at Day 8; PBS vs. Control, P=0.6328). The above statistical results and images indicated that LM injected through interstitial channels has the potential advantage of inhibiting the proliferation of transplanted tumor in BALB/c-nude mice.

### Effective delivery channel for LM

**Figure 4:**
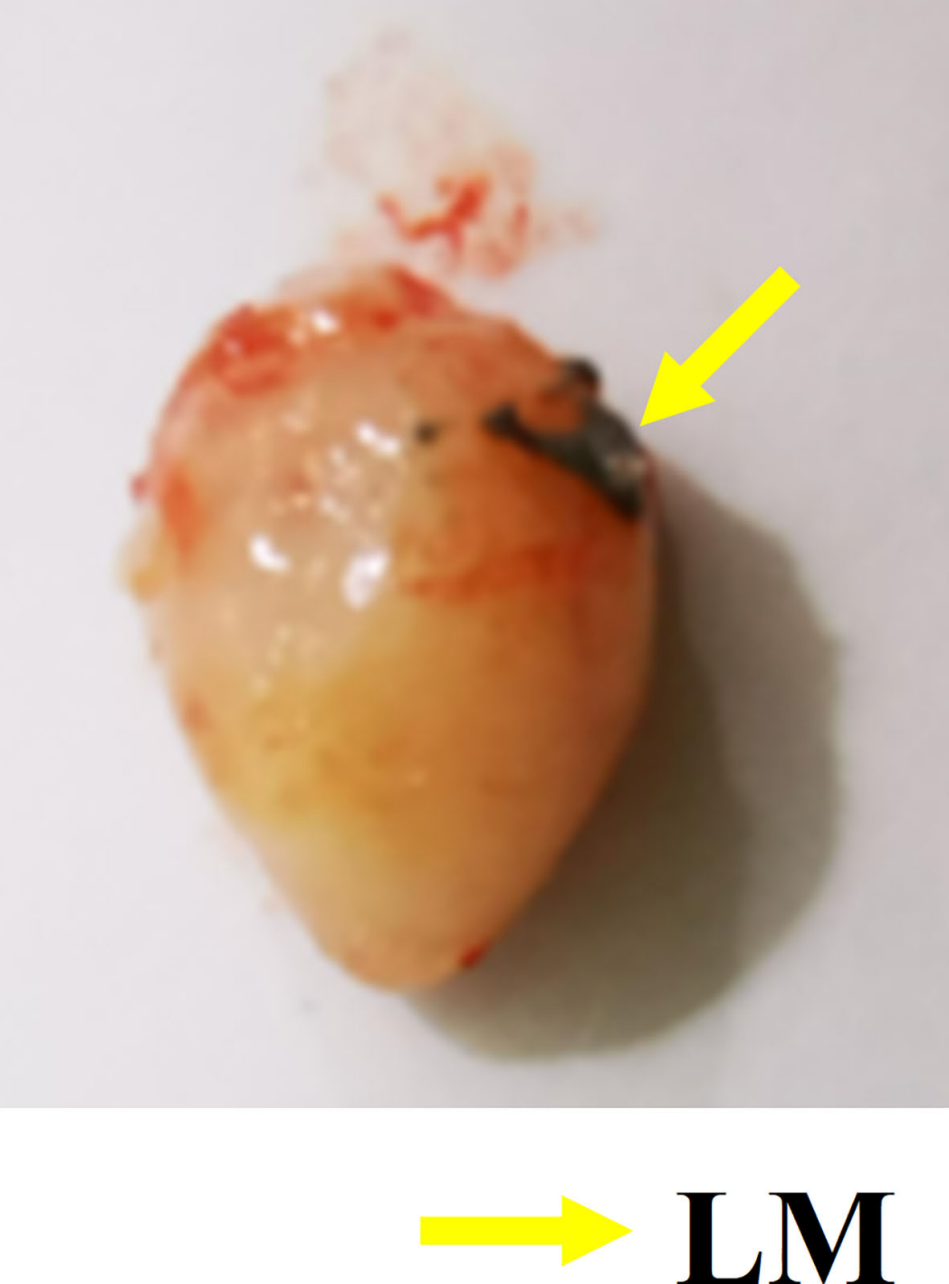
Gross specimen of tumor. The yellow arrow indicates the oxidized LM remaining on the tumor surface. LM, liquid metal.

**Figure 5:**
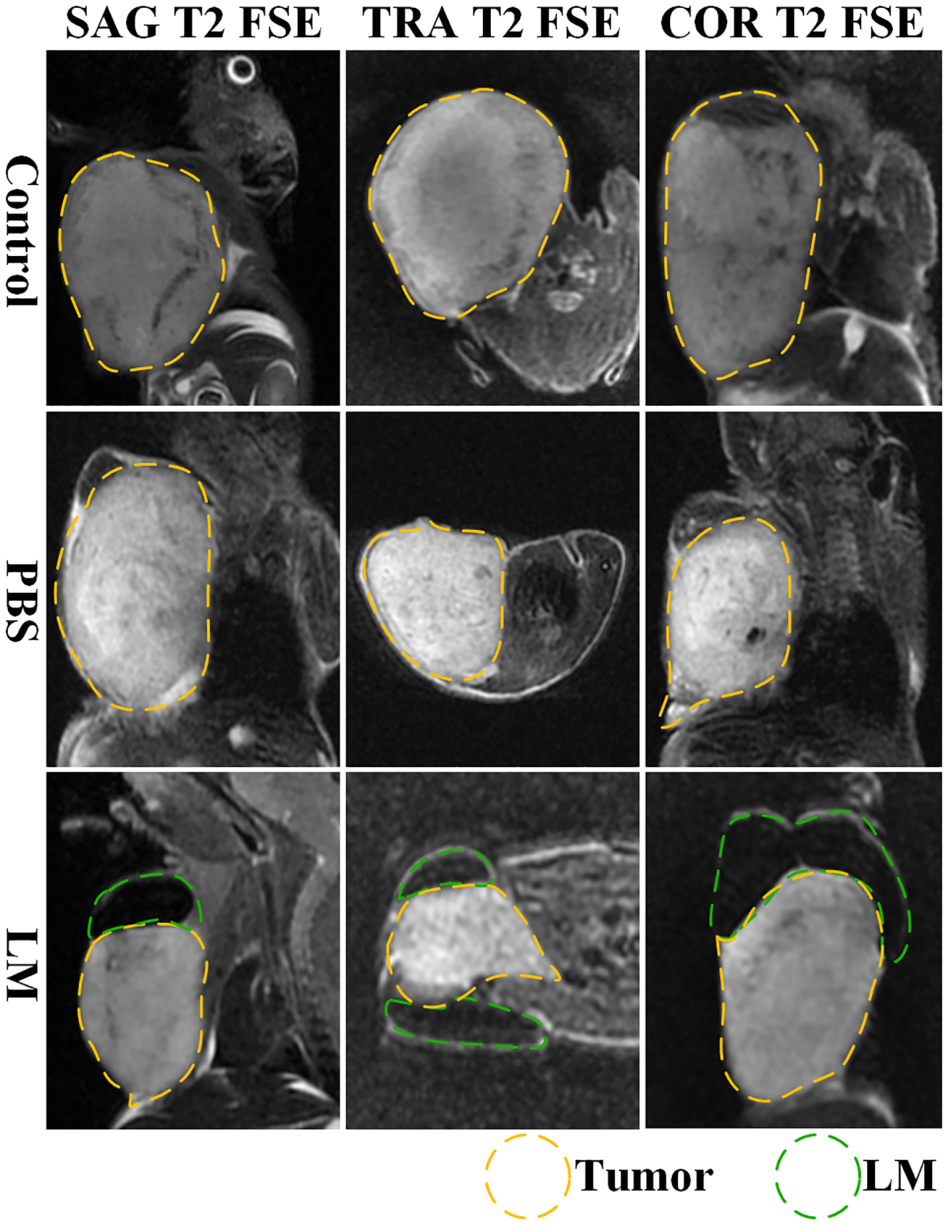
The T2WI tomography images of axial, coronal and sagittal of tumor-bearing mice in each group. Tumor is marked by yellow dotted lines, LM is marked by green dotted lines. SAG T2 FSE, sagittal positions fast spin echo sequences; TRA T2 FSE, axial positions fast spin echo sequences; COR T2 FSE, coronal positions fast spin echo sequences.

Interstitial channels in the limbs of tumor-bearing mice can be used as an effective channel for LM, which can also enhance the differentiation between tumor and normal tissues in MRI images. As shown in **Figure 4**, part of the LM was oxidized after dissection, and only a small fraction remained on the tumor surface, which was smooth. More importantly, the presence of LM provided a clear dividing line between normal and tumor tissue, promising more accurate NRM of the tumor. **Figure 5** shows the T2WI anatomic axial, coronal, and sagittal images in the LM and PBS groups. Three-dimensional profiles of the tumor in each group and the encapsulation effect of LM toward the tumor are displayed in **Figure 5**. It illustrates that LM was close to the tumor boundary and tended to wrap around the tumor as is shown in **Figures 4**, **5**. The coverage area increased with the increase of days, and the edge was clearer than other tissues. In addition, by observing the **Figure 4** dissection of the tumor as well as that in **Figure 5** by upper limb interstitial channel injection of LM wrapped in the tumor area, it further illustrated that the upper extremity interstitial structure is attached directly to the tumor. The interstitial channel may be an effective transmission method, which can be used as a nanodrug transport channel for the treatment of tumors (21). Another possible explanation is that LM injected through the interstitial space tends to encase the tumor surface, preventing the transfer of nutrients necessary for tumor proliferation and contact with other normal tissues and, thus, inhibiting tumor growth. This is consistent with the conclusion of a previous study (22).

Combined with the T2WI anatomic images shown in **Figure 2** and **5**, PBS in the PBS group only existed for 24 h after PBS injection. MRI observation showed that PBS was completely absorbed after 24 h, which was consistent with the normal tumor boundary. PBS in the tumor region was brighter with a blurred boundary, whereas LM in the tumor region was darker with a clear boundary between LM and the tumor. With the growth of the tumor, the interstitial circulation channel of the right upper limb was impaired by the tumor. Therefore, the LM injected into the interstitial flow channel had to flow out at the damaged site, resulting in the accumulation of LM at the boundary of the tumor. On the one hand, it can be seen that the interstitial circulation channel of the right upper limb is connected to the tumor growing under the armpit of the right upper limb, and the interstitial channel has a transport function comparable to blood transport. On the other hand, LM flowing out of the interstitial channel had a wrapping tendency to the tumor over time, which may block and inhibit the growth of the tumor and inhibit the proliferation of the tumor in the wrapping direction. More importantly, the existence of LM provided a clear dividing line between normal tissue and tumor tissue, which was expected to delineate the NRM of the tumor more accurately. The tumor is dissected in **Figure 4**, and it can be clearly seen that LM can separate the tumor from normal tissue, making the tumor boundary clearer; moreover, LM also could be a contrast on MRI.

### Biosafety assessment of LM for tumor-bearing mice

Previous studies indicate that LM possesses mild biosafety and inferior biotoxicity (23, 24). Although significant character differences did not occur in the LM group during the experiment, the side effect of LM in healthy mice cannot be ignored. In this study, histopathological analysis of organ slices and haematology, a serum biochemistry test for tumor-bearing mice, were done after the experiments; the former is used to indicate the pathological lesion of organs in the cellular view and the latter is used to indicate the abnormal level of main indexes.

**Figure 6:**
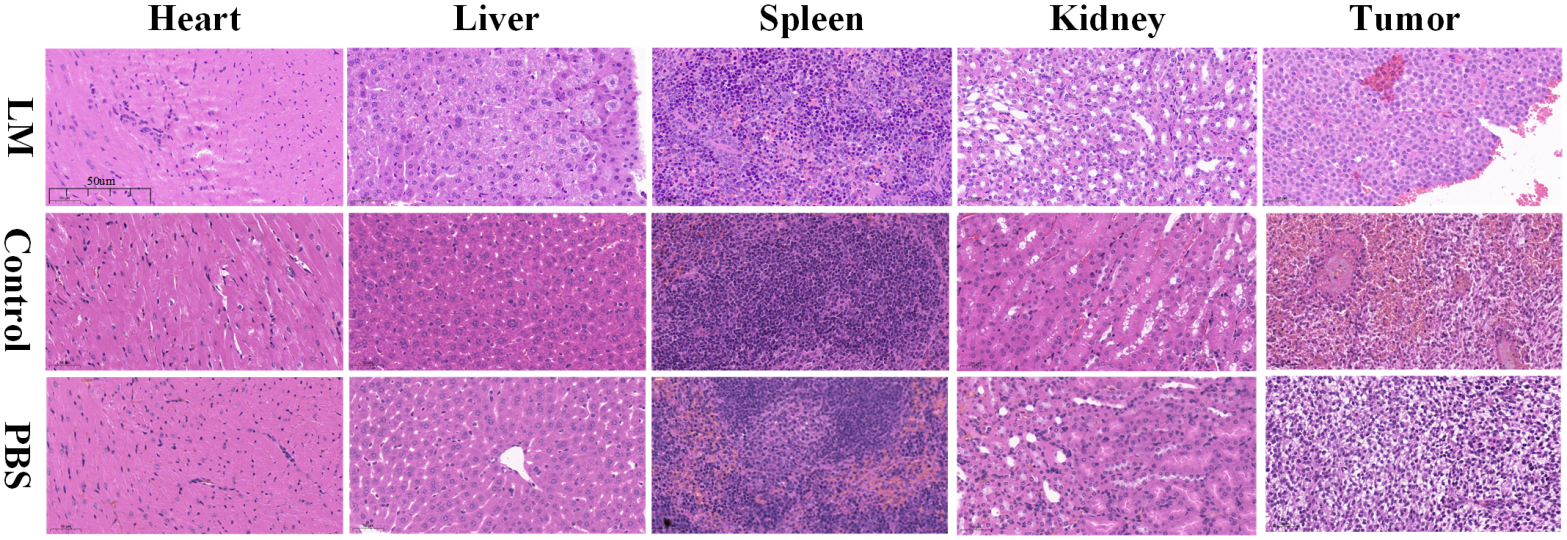
Histopathological images of important parts of tumor-bearing mice including heart, liver, spleen, kidney and tumor in each group. Resolution: 50um. LM, liquid metal; PBS, phosphate buffer saline.

As shown in **Figure 6**, compared with the control and PBS groups, some normal pathological features, such as degeneration, cytoplasmic swelling, and local inflammation, were observed in the heart and liver of the LM group, but there were no obvious differences in spleen and kidney. The histopathological analysis showed that LM injured the major organs of tumor-bearing mice, but the injury degree for organs is acceptable, and the growth of the tumor also confirmed the tolerance of biological safety of tumor-bearing mice toward LM.

**Figure 7:**
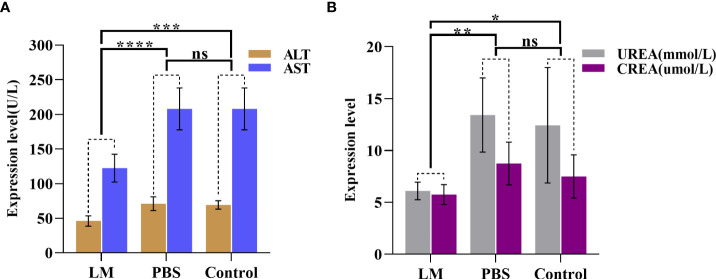
The statistical results of group comparison in serum biochemical tests. **(A)**The expression level of AST and ALT in in three groups. **(B)** The expression level of UREA and CREA in in three groups. LM, liquid metal; PBS, phosphate buffer saline; ALT, Alanine aminotransferase; AST, Aspartate aminotransferase; CREA, creatinine; UREA, carbamide; *, indicates P<0.05; **, indicates P<0.01; ***, indicates P<0.001; ****, indicates P<0.0001, “ns” indicates no significant differences.

AST and ALT are two important transaminases in animals and their expression level in serum will increase after hepatocyte was injured. UREA and CREA are important indicators of animal kidney function (25). **Figure 7** was shown that the expression level histogram of ALT, AST, UREA, CREA of each group, and **Table 3** was the statistical results of serum biochemical tests among three groups and **Table 4** was the results of post-hoc analysis corresponding with **Table 3**. As shown in **Figure 7**, the expression level of ALT, AST, UREA, CREA in LM group was lower than those in the other group and close to normal levels. Meanwhile, the statistical results of **Tables 3**, **4** indicated that there were significant differences among three groups in ALT and AST (ALT: F=12.33, P=0.0026; AST, F=13.22, P=0.0021). The results showed that the liver and kidney indexes of tumor-bearing mice in LM group were within the normal range, although there were significant differences compared with other groups, indicating that LM had certain effects on tumor-bearing mice, but did not cause obvious side effects on liver function and kidney function, and had mild biocompatibility.

**Table 3:** The statistical results of serum biochemical tests among three groups.

	LM Group(n=4)	PBS Group(n=4)	Control Group(n=4)	One-way ANOVA	
	Mean±SD			F value	P value
**ALT**	46.000±7.528	71.000±9.832	69.250±6.021	12.330	0.0026**
**AST**	122.300±20.070	208.000±30.190	211.300±32.020	13.220	0.0021**
**UREA**	6.1000±0.845	13.420±3.576	12.430±5.563	4.252	0.0501
**CREA**	5.750±0.957	8.750±2.062	7.500±2.082	2.868	0.1087

LM, liquid metal; PBS, phosphate buffer saline; ALT, Alanine aminotransferase; AST, Aspartate aminotransferase; CREA, creatinine; UREA, carbamide; SD, standard deviation; n, number of sample; **, indicates P<0.01.

**Table 4:** The statistical results of post-hoc analysis in serum biochemical tests.

	LM vs. PBS	LM vs. Control	PBS vs. Control
	Adjusted P value		
**ALT**	0.0041**	0.0064**	0.9483
**AST**	0.0048**	0.0038**	0.9852
**UREA**	0.0468*	0.1032	0.9302
**CREA**	0.0941	0.3853	0.5990

LM, liquid metal; PBS, phosphate buffer saline; ALT, Alanine aminotransferase; AST, Aspartate aminotransferase; CREA, creatinine; UREA, carbamide; Adjusted P value, the statistical result of Tukey's multiple comparisons test; *, indicates P<0.05; **, indicates P<0.01.

**Figure 8:**
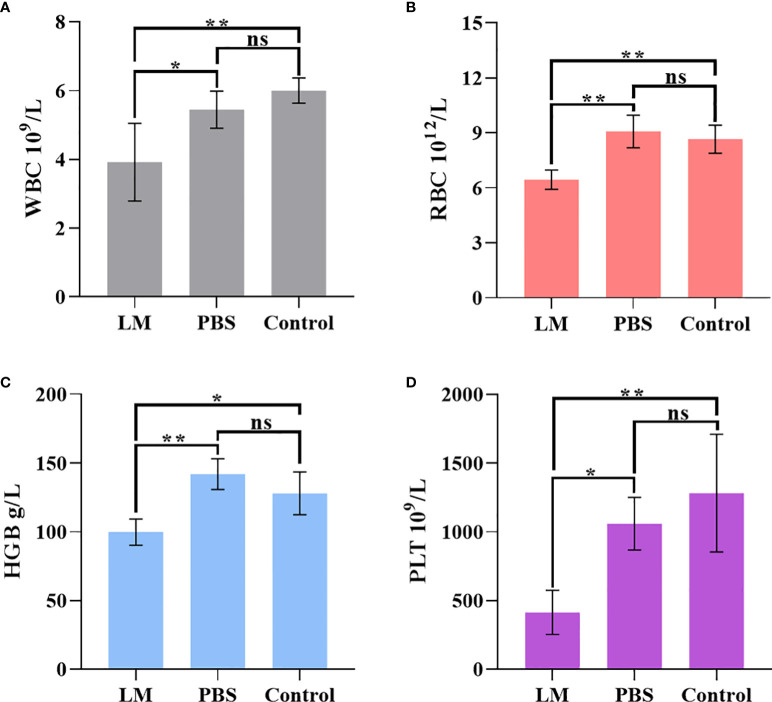
The statistical results of group comparison in haematology tests. **(A-D)** represents the expression level of WBC, RBG, HGB, PLT in three groups, respectively. LM, liquid metal; PBS, phosphate buffer saline; WBC, blood cell count; RBC, Red blood cell count; HGB, Hemoglobin; PLT, Platelet count; *, indicates P<0.05; **, indicates P<0.01. ns, indicates no significant differences.

WBC, RBC, HGB, PLT are the important components of maintaining hematopoietic and immune function (26). **Figure 8** was shown that the expression level histogram of WBC, RBC, HGB, PLT of each group. And, **Table 5** was the statistical result of hematology tests among three groups and **Table 6** was the result of post-hoc analysis corresponding with **Table 5**. As shown in **Figure 8**, the expression level of WBC, RBC, HGB, PLT in LM group was lower than other group, but were close to lower limit of normal levels. Meanwhile, the statistical results of **Tables 5**, **6** indicated that there were differences among three groups (WBC, F=8.154, P=0.0095; RBC, F=14.4, P=0.0016; HGB, F=12.09, P=0.0028; PLT, F=9.918, P=0.0053). Serum test results showed that the immune system and hematopoietic function of tumor-bearing mice in the LM group were lower than those in the PBS group and the control group, which may be caused by the slight damage caused by LM at the cellular level to the functional organs of tumor-bearing mice. Further research on LM-based nanomaterials may reduce these differences.

**Table 5:** The statistical results of haematology tests among three groups.

	LM Group(n=4)	PBS Group(n=4)	Control Group(n=4)	One-way ANOVA	
	Mean±SD			F value	P value
**WBC**	3.920±1.132	5.448±0.539	6.000±0.369	8.154	0.0095**
**RBC**	6.440±0.526	9.073±0.891	8.650±0.772	14.400	0.0016**
**HGB**	99.75±9.570	142.00±11.22	128.00±15.56	12.090	0.0028**
**PLT**	414.30±160.50	1060.00±191.10	1282.00±428.30	9.918	0.0053**

LM, liquid metal; PBS, phosphate buffer saline; WBC, blood cell count; RBC, Red blood cell count; HGB, Hemoglobin; PLT, Platelet count; SD, standard deviation; n, number of sample; **, indicates P<0.01.

**Table 6:** The statistical results of post-hoc analysis in haematology tests.

	LM vs. PBS	LM vs. Control	PBS vs. Control
	Adjusted P value		
**WBC**	0.0448*	0.0091**	0.5746
**RBC**	0.0019**	0.0059**	0.7112
**HGB**	0.0024**	0.0254*	0.2947
**PLT**	0.0270*	0.0052**	0.5386

LM, liquid metal; PBS, phosphate buffer saline; WBC, blood cell count; RBC, Red blood cell count; HGB, Hemoglobin; PLT, Platelet count; Adjusted P value, the statistical result of Tukey's multiple comparisons test; *, indicates P<0.05; **, indicates P<0.01.

### Excellent detectable medium for MAT

**Figure 9:**
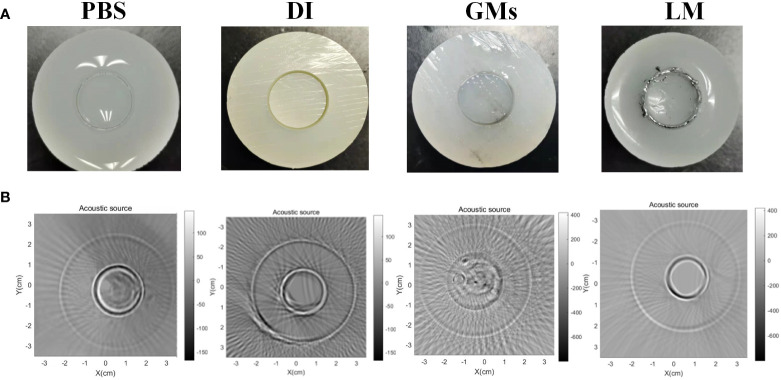
The cylindrical agar-body with different fill materials and correspondent MAT images. **(A)** PBS, DI, Gallium micropaticels(GMs), LM filled the 1mm clearance of cylindrical agar-body. **(B)** The correspondent MAT images of cylindrical agar-body with various materials. PBS, phosphate buffer saline; DI, Deionized water; GMs, Gallium-based ally microparticles; LM, liquid metal.

As a new conductivity imaging method, MAT, has the advantages of the high contrast of electromagnetic imaging and high resolution of ultrasonic imaging (27–30), and it has a good prospect for the early diagnosis of tumors. The change of conductivity is earlier than the structural change in the early development of tumors, so it is expected to image tumors in the early stages. However, in the detection of living animals, considering the limitation of safe current, the magnetoacoustic signal detected in MAT is very weak, and its imaging resolution is related to the detection sensitivity of the weak signal. Especially in the detection of living animals, the enhancement medium with high conductivity in the tumor area is an effective means for MAT to improve the image quality (31–33). Considering the wrapping effect of LM on the tumor boundary and the high conductivity characteristics of LM, we use LM as the functional material for enhancing the conductivity of the tumor area, and try to enhance the amplitude of the weak MAT detection signal, so as to improve the resolution of the MAT image (34–37). To verify the feasibility of LM as a functional material with enhanced conductivity, **Figure 9** shows the comparison of MAT under different liquid filling materials. In **Figure 9**, a 1-mm circular clearance is used to simulate the boundary of the tumor, agar-body with 0.2S/m 0.2s/m represents normal tissue, and various liquid filling materials (PBS, DI, GMS, LM) play the role of covering the tumor. The color scale value in **Figure 9** represents the signal strength. To highlight the enhancement effect of LM, the MAT signal measured under the same excitation parameters is used to reconstruct the sound source of the original signal. Compared with the MAT images of other materials, the signal intensity and resolution of LM are significantly higher than those of other materials.

**Figure 10:**
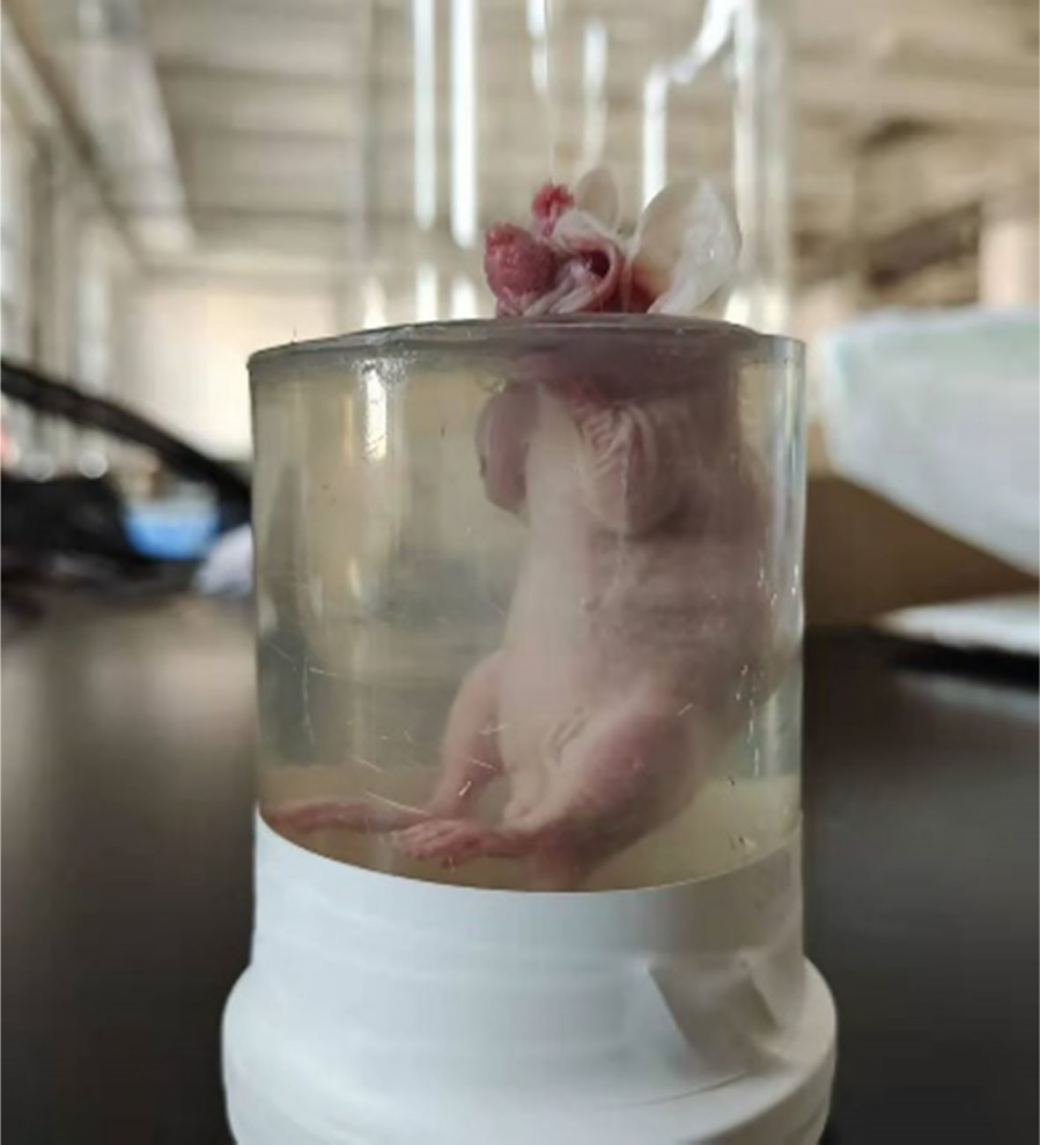
The model of mouse for Magnetoacoustic experiment.

**Figure 11:**
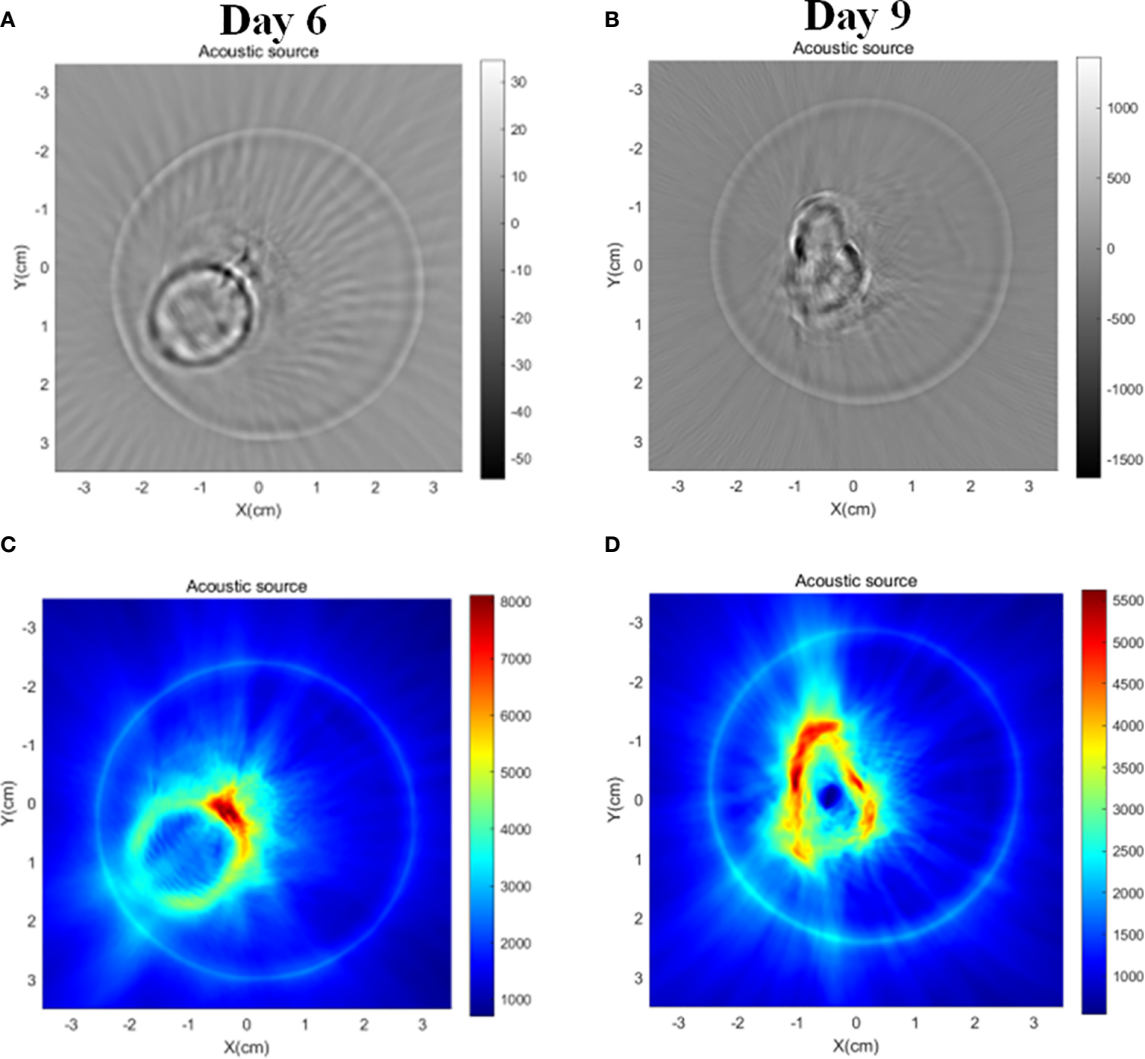
MAT images of tumor-bearing mice in LM group on day 6 and day 9, respectively. **(A)** MAT images of tumor mice on day 6 **(B)** MAT images of tumor mice on day 9. **(C)** Magneto-acoustic signal intensity distribution images of tumor mice on day 6, and **(D)** magneto-acoustic signal intensity distribution images of tumor mice on day 9. The red area in the image represents the LM distribution area.

As shown in **Figure 10**, the tumor mice and the cylindrical glass container used to support and hold the mice were prepared before the *in vivo* tumor magnetoacoustic tomographic experiment. First, the mice were anesthetized and placed in a cylindrical glass container for fixation. At the same time, the liquid agar gel kept at 37°C was poured into a glass container. After approximately 10 min, the liquid agar gel solidified and adhered to the mice to form a test sample. Then, the mice, together with the solidified agar gel, were taken out from the glass container for magnetoacoustic tomography. The conductivity of agar gel in **Figure 10** is 0.1s/m. In the MAT experiment, the direction of the stimulated current density in the mice is circumferential, so the tomographic image of the mice can be obtained by detecting the magnetoacoustic signal in a circle around the mice abdomen. **Figure 11** shows the sectional images and LM distribution images on the 6th and 9th days after LM injection, respectively. The regular large ring in **Figure 11** (A) and (C) is the boundary of the fixed imitation, the inside is the sectional image of mice, and the red area in (B) and (D) is the liquid metal distribution area. The contour and boundary of tumors at different stages can be clearly distinguished in **Figure 11**, which fully demonstrates the enhancement effect of LM on the weak MAT signal.

## Conclusion

In summary, we used the interstitial space as a liquid transport channel. By injecting LM into the interstitial space of the right upper limb of tumor bearing mice, affected by the negative pressure effect of interstitial space and blocked and destroyed by the tumor growing near the right upper limb, LM was passively introduced into the tumor area of tumor-bearing mice. LM partially separated the tumor from its nearby healthy tissue and further effectively inhibited the growth rate of the tumor. The observation and analysis of the anatomical structure and MRI T2 scanning images of mice injected with LM also confirmed that interstitial channels may be an effective transport path for liquid substances or future nano drug carriers. In addition, using MRI, we found that LM has the potential to delineate tumor boundaries and has the advantages of MRI and MAT enhanced-contrast imaging. The MRI T2 scan can outline the exact size and location of the tumor, and the MAT can obtain the functional information corresponding to the changes in electrical properties during tumor growth. At present, tumor surgery is trying to find a new method to display the NRM of the tumor. The two imaging modes are expected to obtain more effective NRM, which provides a new idea for tumor surgery. This discovery provides a new idea for tumor surgery. Furthermore, histopathological analysis and hematological tests were carried out on LM mice with a scientific and rigorous attitude, and the results showed that although LM existed *in vivo* for a long time and caused some effects and damage to mouse organs, it had no obvious toxicity and side effects compared with tumor mice growing at the same time.

## Data availability statement

The original contributions presented in the study are included in the article/supplementary material. Further inquiries can be directed to the corresponding authors.

## Ethics statement

This study was reviewed and approved by Biomedical Research Ethics Review Committee of Institute of Electrical Engineering, Chinese Academy of Sciences. Written informed consent was obtained from the owners for the participation of their animals in this study.

## Author contributions

Conceptualization, HX, GM, FX, GL, CL and AY. Methodology, HX, GM. Software, CL, JL and AY. Validation, WZ, JL and AY. Formal analysis, YL, HZ and WZ. Investigation, YL, FX and HX. Data curation, CL and AY. Writing—original draft preparation, CL and AY. Writing—review and editing, HX, GM, GL, HZ, CL, AY, WZ, JL, FX and YL. Supervision, HX and GM. Project administration, HX. Funding acquisition, HX. All authors contributed to the article and approved the submitted version.

## Funding

This work was supported by grants from the Natural Science Foundation of Beijing (No.3214064, No.7212210), the Natural Science Foundation of China (No.51937010, No.81971585, No.82271953,No.82271953), the Beijing Science and Technology Commission Project (No.Z181100003818006), the scientific instrument Project of Chinese Academy of Sciences (No. ZD-KYYQ20190002), the National Key Research and Development Program of China (Nos. 2020YFC2003903), the Guangzhou Science and technology planning project (202103010001), and Beijing Municipal Science and Technology Project (Z211100003521009).

## Conflict of interest

The authors declare that the research was conducted in the absence of any commercial or financial relationships that could be construed as a potential conflict of interest.

The reviewer DD declared a shared affiliation with the authors CL, AY, GL, WZ, JL, GM, HX to the handling editor at the time of review.

## Publisher’s note

All claims expressed in this article are solely those of the authors and do not necessarily represent those of their affiliated organizations, or those of the publisher, the editors and the reviewers. Any product that may be evaluated in this article, or claim that may be made by its manufacturer, is not guaranteed or endorsed by the publisher.
